# Supplementary material for: FlashPCR: Revolutionising qPCR by Accelerating Amplification through Low ∆T Protocols
Source: Int J Mol Sci. 2024 Feb 28;25(5):2773. doi: 10.3390/ijms25052773 (PMC10932470; doi:10.3390/ijms25052773)
Supplement: Supplementary file 1 [file ijms-25-02773-s001.zip › Final Supp Fig .pdf]

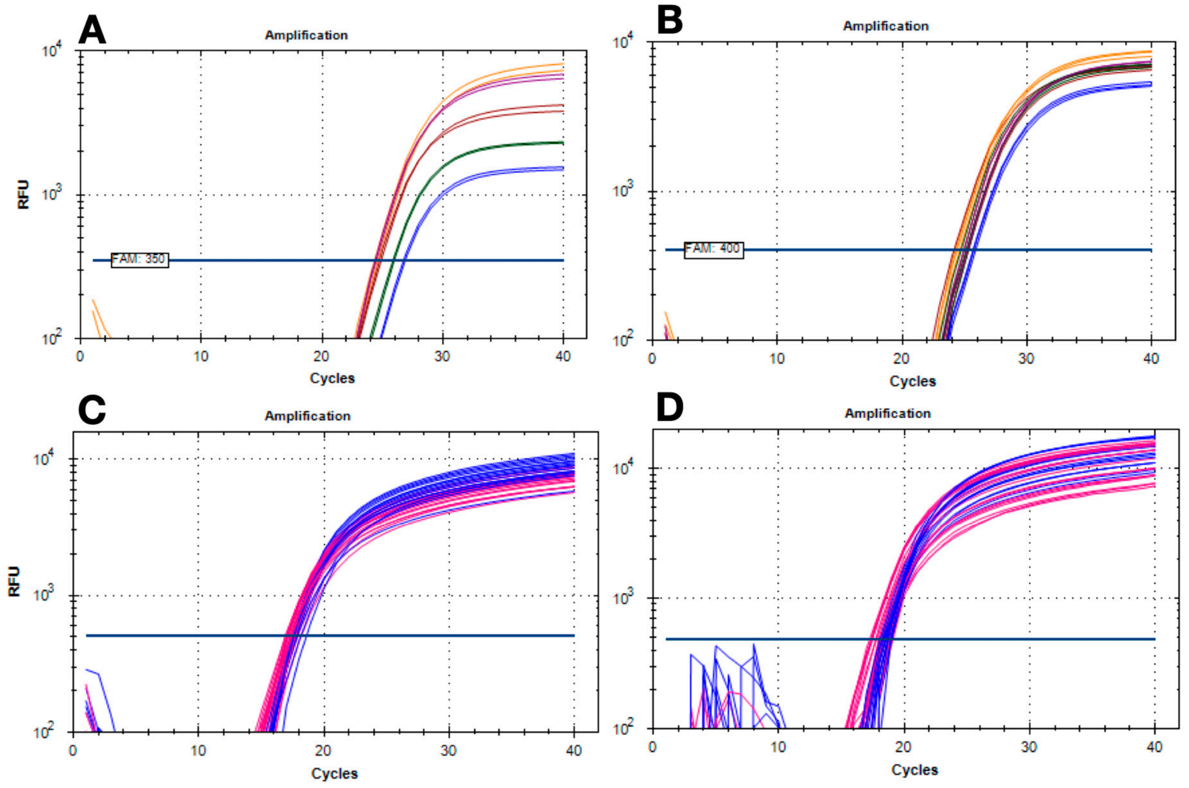

Figure S1. Establishing optimal probe, primer and MyTaq concentrations with B47. Horizontal lines show the position of the threshold used to calculate Cq values. **A.** Amplification plots recorded with a range of probe concentrations using the standard fast protocol P1 (0.05µM blue, 0.1µM green, 0.2µM brown, 0.3µM purple, 0.4µM orange). **B.** Amplification plots recorded with a range of primer concentrations using the standard fast protocol (P1) (1.5µM orange, 1µM purple, 0.75µM brown, 0.5µM green, 0.25µM blue). **C.** Amplification plots recorded with 0.06 (pink) and 0.12U (blue) of MyTaq per 2.5µL reaction across the denaturation gradient protocol P2. **G.** Amplification plots recorded with 0.06 (pink) and 0.12U (blue) of MyTaq per 2.5µL reaction across the polymerisation gradient protocol P3. All Cq values are listed in the Supplementary data file.

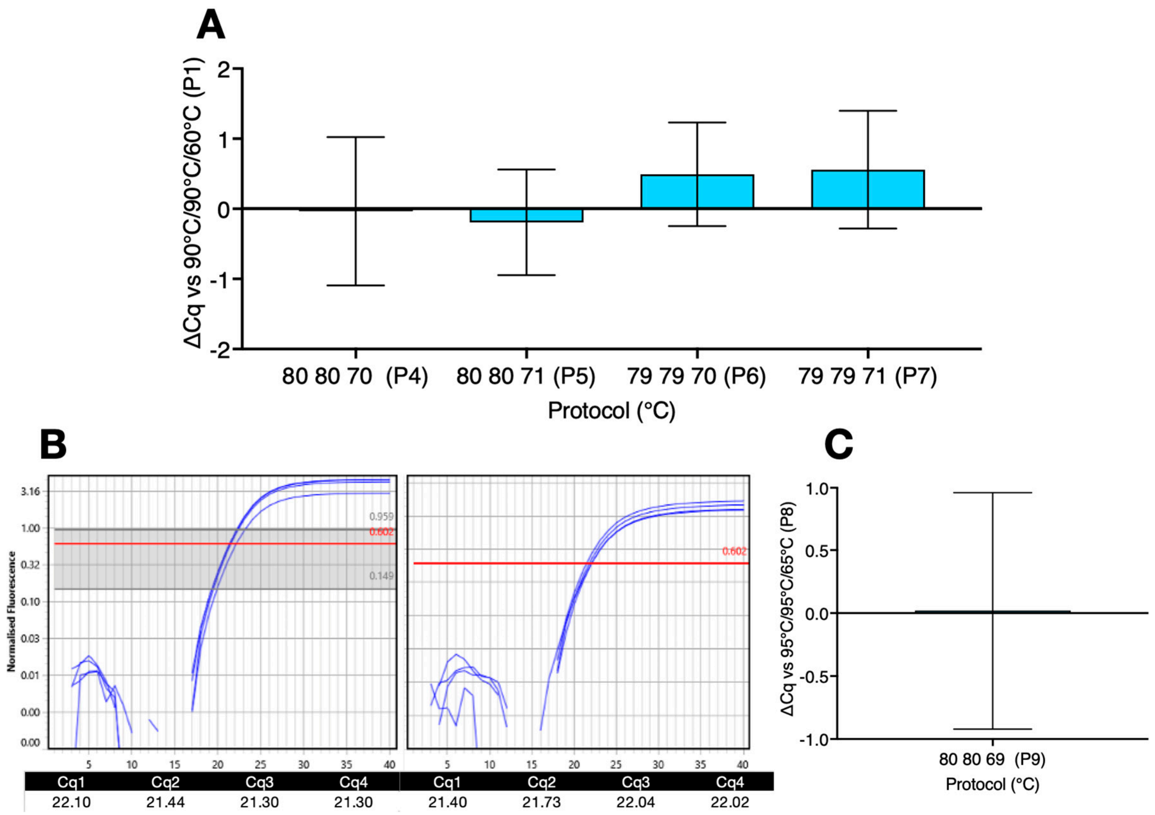

Figure S2. Amplification of SARS-CoV-2 cDNA with assay CoV-E, B47 and MyTaq run on BioRad CFX Connect or BMS Mic qPCR instruments. All Biorad Cq values are listed in the Supplementary data file. **A.** ΔCq values (±95% CI) recorded at the indicated cycling temperatures versus protocol P1 for the combined runs. **B.** Amplification plots and Cq values for standard (P8) and fast protocols (P9) on the BMS Mic. Horizontal lines show the position of the threshold used to calculate Cq values. **C.** ΔCq values (±95% CI) recorded for P9 compared to P8.

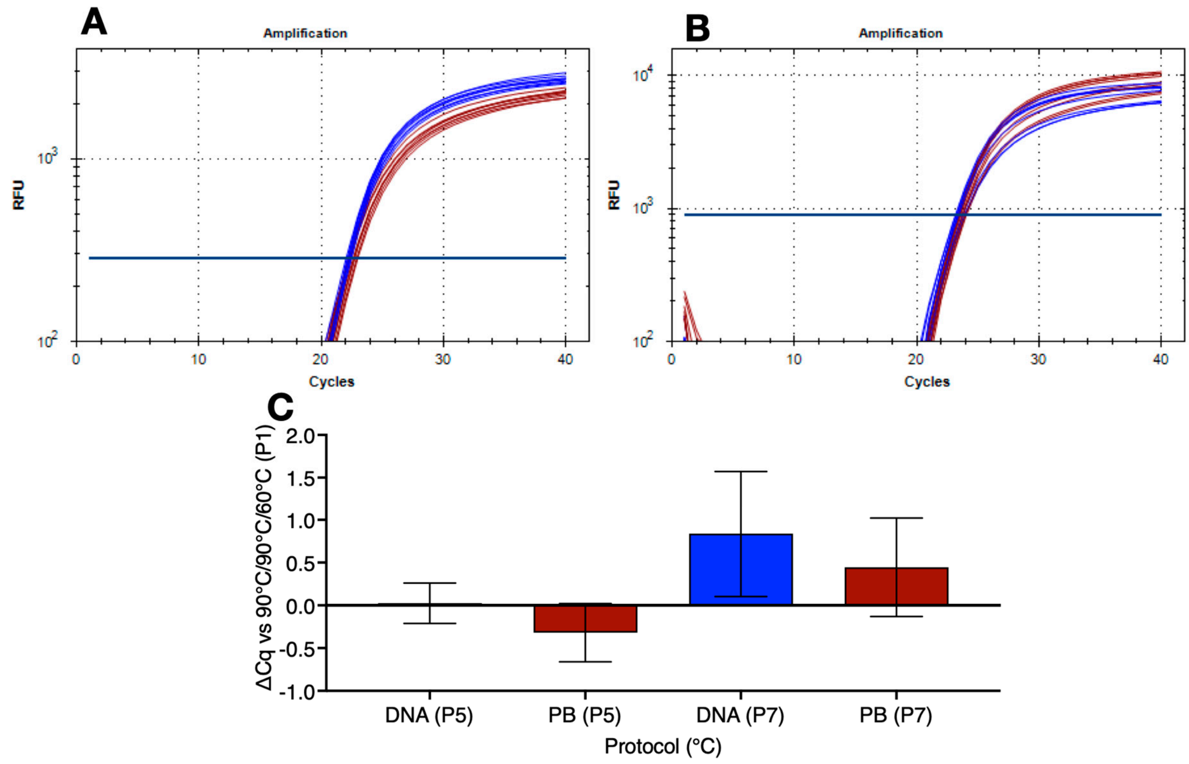

Figure S3. Amplification of SARS-CoV-2 PCR amplicons with DNA primers (blue) and Pentabase primers (PB, brown), Probe G and B47. **A.** Amplification plots recorded on a denaturation gradient using protocol P10. Horizontal lines show the position of the threshold used to calculate Cq values. **B.** Amplification plots recorded on a polymerisation gradient using protocol P3. Horizontal lines show the position of the threshold used to calculate Cq values. **C.** ΔCq values (±95% CI) recorded at the indicated cycling temperatures versus protocol P1 for the DNA (blue) and Pentabase (brown) primers. All Cq values are listed in the Supplementary data file.

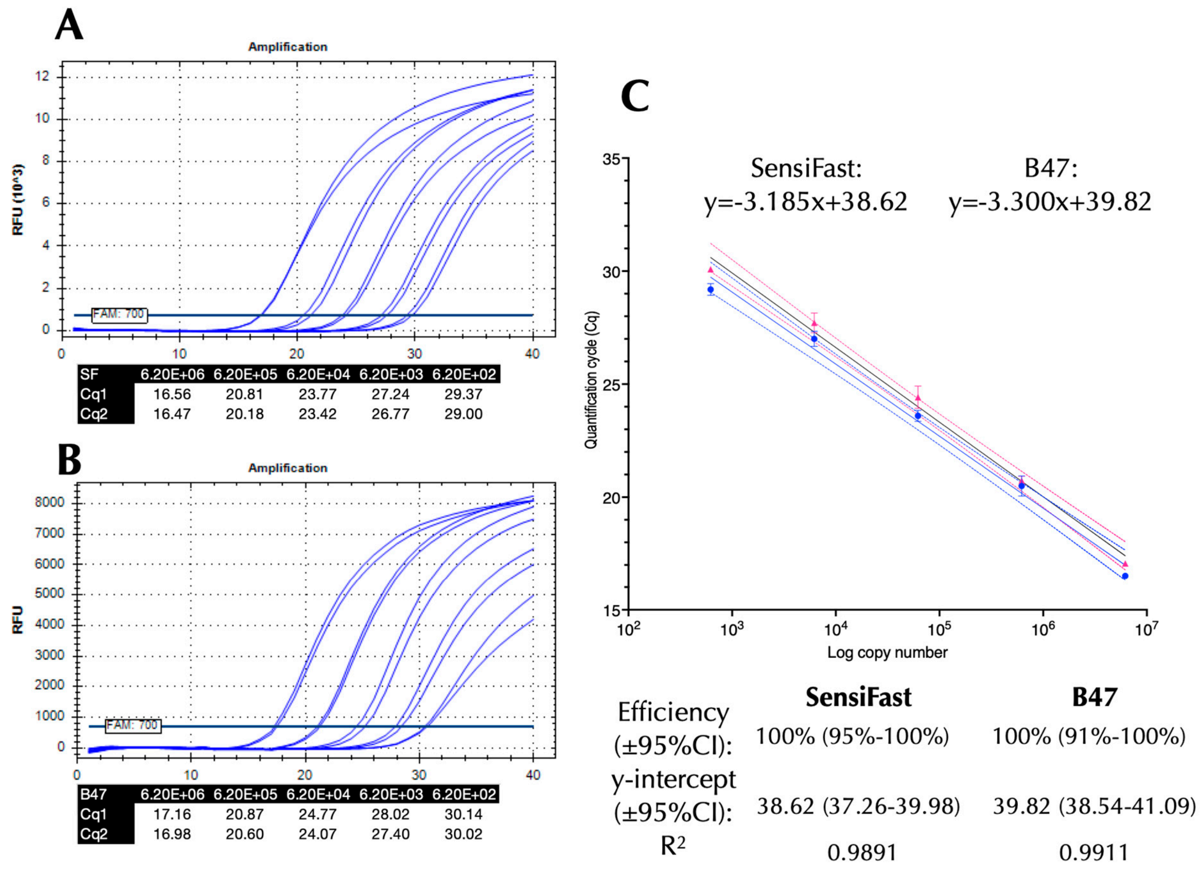

Figure S4. Linearity and efficiency of 4E-2 (G) qPCR assay. **A.** Amplification plots and Cq values recorded under standard PCR conditions with SensiFast master mix. Horizontal lines show the position of the threshold used to calculate Cq values. **B.** Amplification plots and Cq values recorded under reduced ΔT conditions with buffer 47. Horizontal lines show the position of the threshold used to calculate Cq values. **C.** Standard curves for SensiFast (blue) and B47 (pink) with 95% CI.

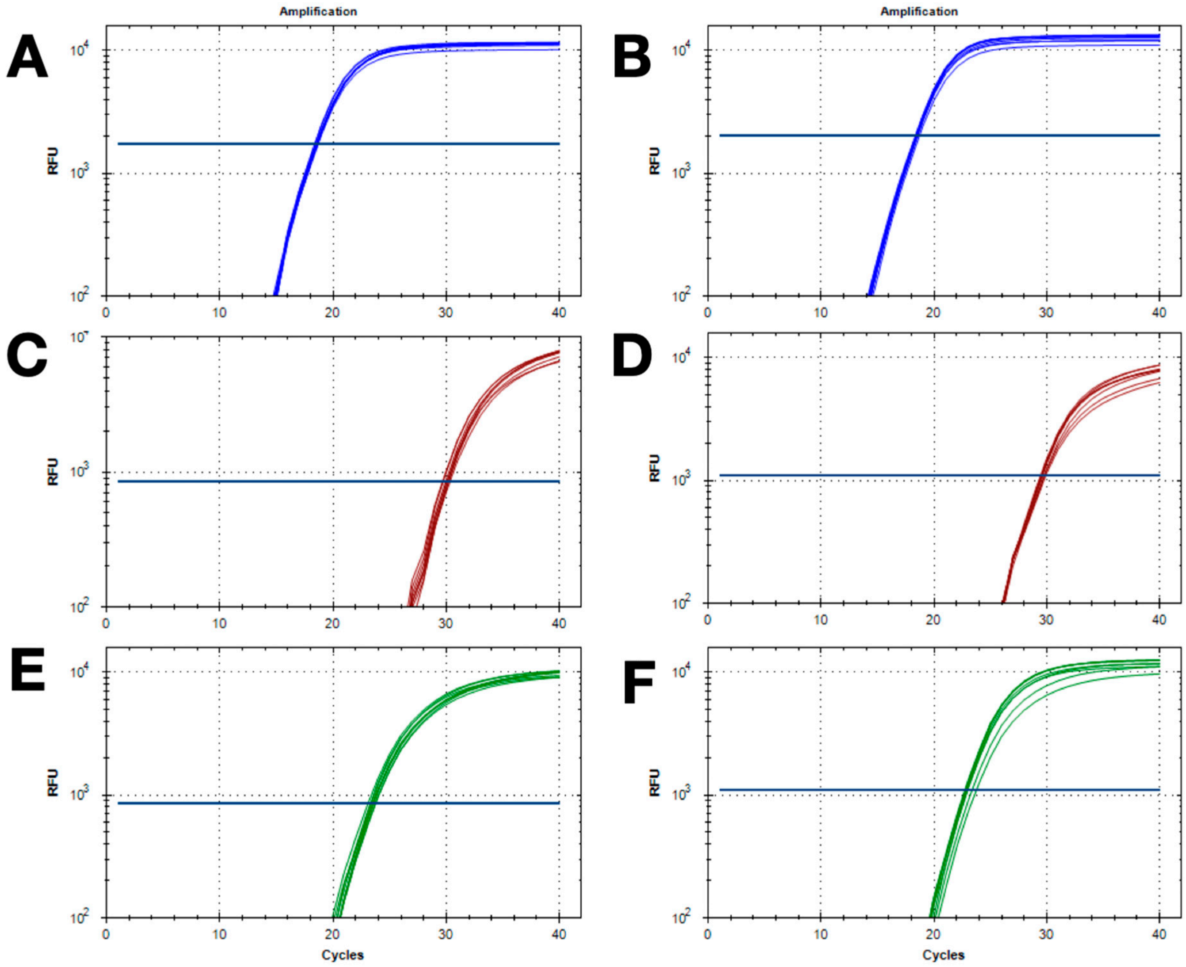

Figure S5. Amplification of pathogen gDNA on denaturation and polymerisation gradients with B27 on a BioRad CFX Connect. Horizontal lines show the position of the threshold used to calculate Cq values. **A.** Amplification plots and Cqs recorded with *C. auris* gDNA on a denaturation gradient using protocol P12. **B.** Amplification plots and Cqs recorded with *C. auris* gDNA on a polymerisation gradient using protocol P13. **C.** Amplification plots and Cqs recorded with *A. fumigatus* gDNA on a denaturation gradient using protocol P12. **D.** Amplification plots and Cqs recorded with *A. fumigatus* gDNA on a polymerisation gradient using protocol P13. **E.** Amplification plots and Cqs recorded with *Acanthamoeba castelanii* gDNA on a denaturation gradient using protocol P12. **F.** Amplification plots and Cqs recorded with *Acanthamoeba castelanii* gDNA on a polymerisation gradient using protocol P13. All Cq values are listed in the Supplementary data file.

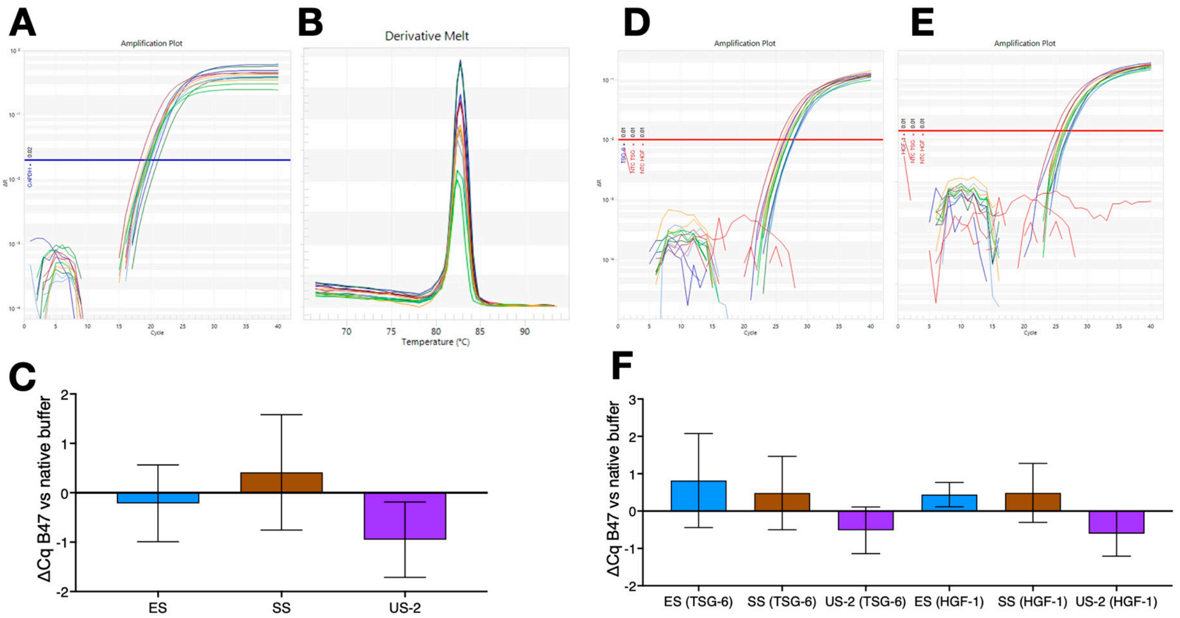

Figure S6. 2-step RT-qPCR analysis of human breast cancer with mRNA reverse transcribed using three RTs in buffer 47 or their native buffers on a Hybaid PrimePro 48 qPCR instrument. **A.** GAPDH amplification plots recorded for each of the mRNA synthesised with native buffers or buffer 47 (ES: native buffer dark blue, B47 light blue; SSIV native buffer brown, B47 orange; US-2 native buffer dark green, B47 light green). Horizontal line shows the position of the threshold used to calculate Cq values. **B.** Corresponding melt curves. **C.** Plot of ΔCq values (±95% CI) recorded for each of the mRNA samples reverse transcribed with buffer 47 versus the Cqs recorded from mRNA samples reverse transcribed with native buffers. **D.** TSG-6 amplification plots and Cq values recorded for each of the cDNAs synthesised with native buffers or buffer 47 and amplified using Luna master mix. Horizontal line shows the position of the threshold used to calculate Cq values. (ES: native buffer dark blue, B47 light blue; SSIV native buffer brown, B47 orange; US-2 native buffer dark green, B47 light green) **E.** HGF-1 amplification plots and Cq values recorded for each of the cDNAs synthesised with native buffers or buffer 47 and amplified using Luna master mix. Horizontal line shows the position of the threshold used to calculate Cq values. (ES: native buffer dark blue, B47 light blue; SSIV native buffer brown, B47 orange; US-2 native buffer dark green, B47 light green). **F.** Plot of ΔCq values (±95% CI) recorded for each of the mRNA samples reverse transcribed with buffer 47 versus the Cqs recorded from mRNA samples reverse transcribed with native buffers. All Cq values are listed in the Supplementary data file.

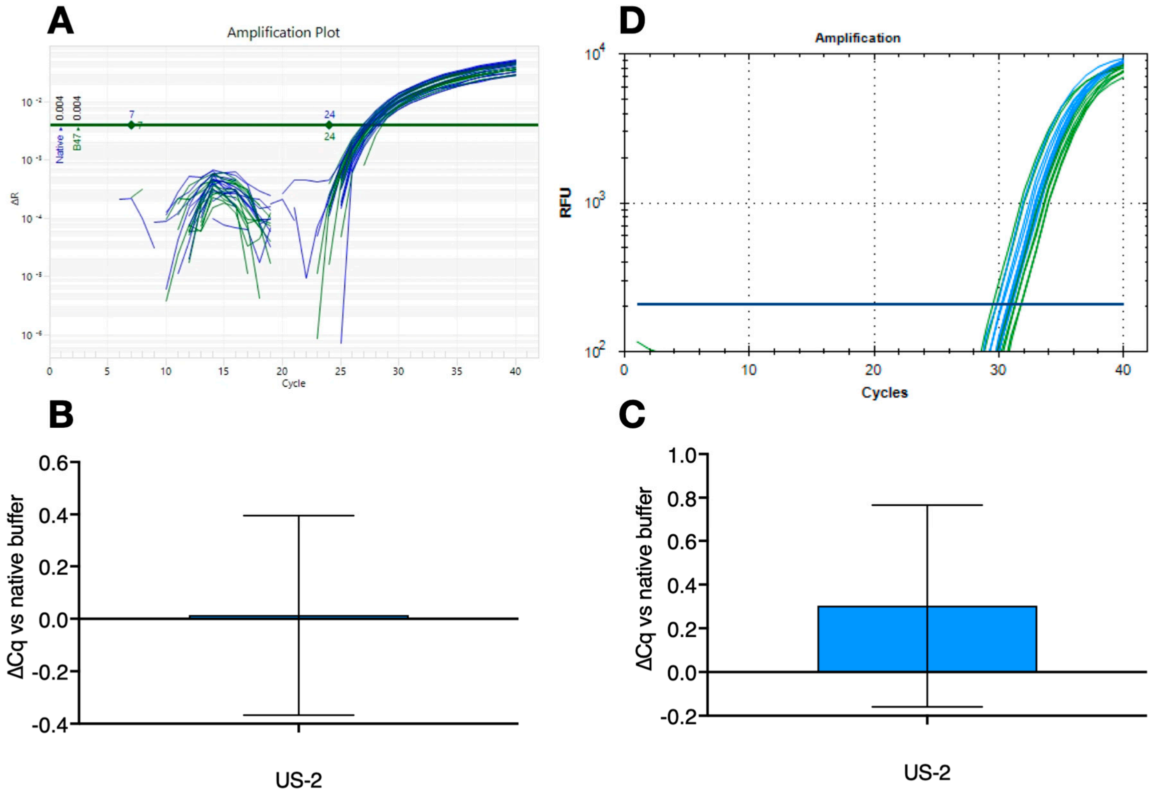

Figure S7. 2-step RT-qPCR reactions with SARS-CoV-2 gRNA reverse transcribed with native RT buffers or B47 and using the CoV-E assay. **A.** Amplification plots recorded for mRNA reverse transcribed with EpiScript (ES) in its native buffer (n=7) (dark blue) or B47 (n=7) (dark green) and amplified with SensiFast on a Hybrid PrimePro 48 instrument. Horizontal lines show the position of the threshold used to calculate Cq values. **B.** ΔCq values (±95% CI) recorded for the combined mRNA samples reverse transcribed with buffer 47 versus the Cqs recorded from mRNA samples reverse transcribed with native buffers. **C.** Amplification plots recorded for mRNA reverse transcribed with UltraScript-2 (US-2) in its native buffer (n=4) (light blue) or B47 (n=4) (light green) and amplified with B47 on a BioRad Opus instrument. Horizontal lines show the position of the threshold used to calculate Cq values. **D.** ΔCq values (±95% CI) recorded for the combined mRNA samples reverse transcribed with buffer 47 versus the Cqs recorded from mRNA samples reverse transcribed with native buffers. All Cq values are listed in the Supplementary data file.

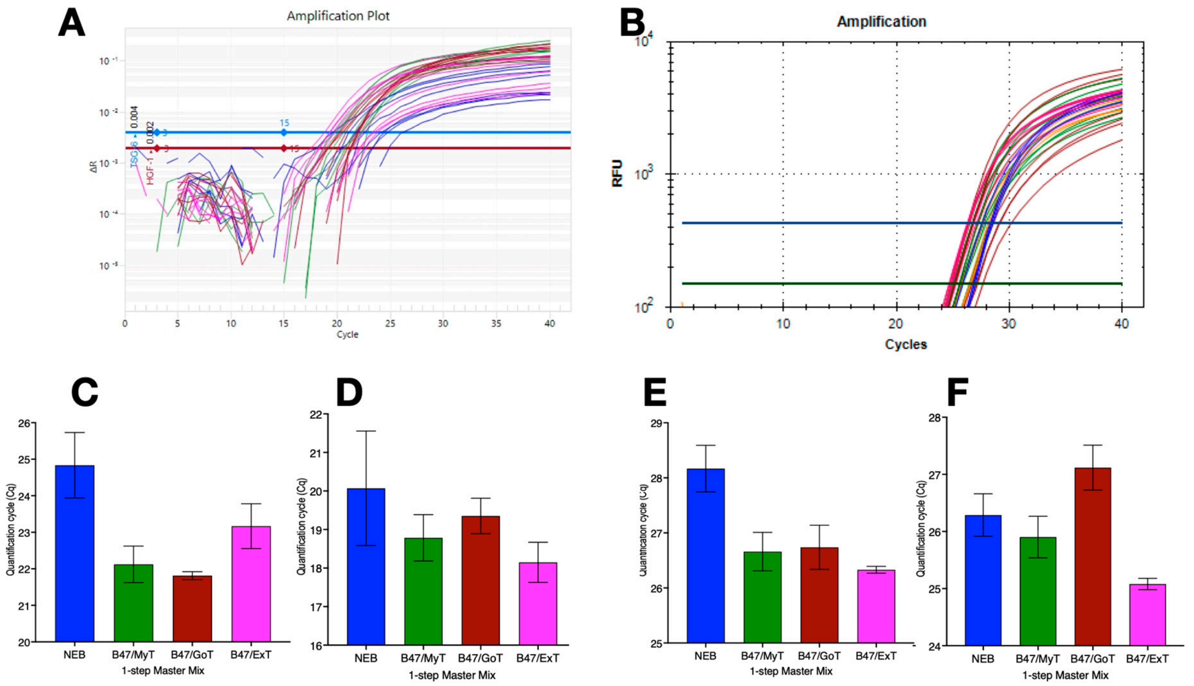

Figure S8. 1-step RT-qPCR reactions with human breast cancer mRNA using NEB Luna (blue) or EpiScript/B47 and MyTaq (green), GoTaq (brown) or ExTaq (pink) 1-step master mixes. **A.** Amplification plots for TSG-6 (FAM) and HGF-1 (HEX) on the PrimePro 48. Horizontal lines show the position of the thresholds used to calculate Cq values. **B.** Amplification plots and Cq values for TSG-6 (FAM) and HGF-1 (HEX) on the BioRad CFX Connect. Horizontal lines show the position of the thresholds used to calculate Cq values. **C.** Plot of Cq values (±95% CI) for TSG on the PrimePro 48. **D.** Plot of Cq values (±95% CI) for HGF-1 on the PrimePro 48. **E.** Plot of Cq values (±95% CI) for TSG on the BioRad CFX Connect. **F.** Plot of Cq values (±95% CI) for HGF-1 on the BioRad CFX Connect. All Cq values are listed in the Supplementary data file.

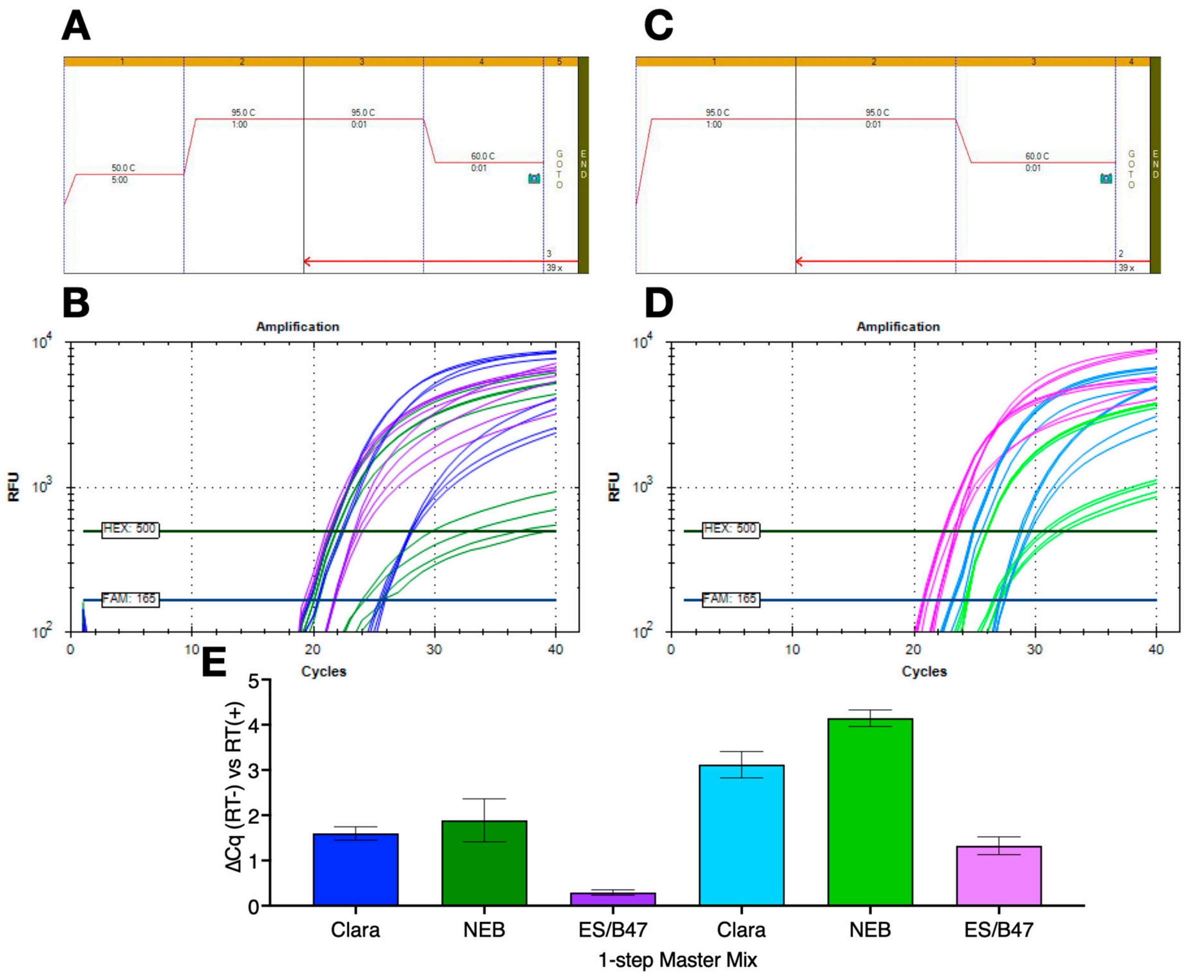

Figure S9. Comparison of 1-step RT-qPCR reactions carried out targeting human breast cancer RNA using duplex TSG-6/HGF-1 assays with and without a dedicated RT step. **A.** 1-step RT-qPCR protocol (R2) with dedicated RT step run on a BioRad CFX Connect. **B.** Amplification plots and Cq values for PCRBio Clara (dark blue), NEB Luna (dark green) and B47/ES/MyTaq (purple) 1-step master mixes**.** Horizontal lines show the position of the thresholds used to calculate Cq values. **C.** 1-step RT-qPCR protocol R3 without a dedicated RT step. Horizontal lines show the position of the threshold used to calculate Cq values. **D.** Amplification plots and Cq values for PCRBio Clara (light blue), NEB Luna (light green) and B47/ES/MyTaq (pink) 1-step master mixes using the modified protocol. **E.** ΔCq values (±95% CI) of the TSG-6 (dark blue, dark green and purple) and HGF-1 (light blue, light green and pink) reactions carried out with protocol R3 versus protocol R2. All Cq values are listed in the Supplementary data file.
